# An Open-Source Label Atlas Correction Tool and Preliminary Results on Huntingtons Disease Whole-Brain MRI Atlases

**DOI:** 10.3389/fninf.2016.00029

**Published:** 2016-08-03

**Authors:** Jessica L. Forbes, Regina E. Y. Kim, Jane S. Paulsen, Hans J. Johnson

**Affiliations:** ^1^Department of Psychiatry, University of IowaIowa City, IA, USA; ^2^Department of Biomedical Engineering, University of IowaIowa City, IA, USA; ^3^Department of Neurology, Carver College of Medicine, University of IowaIowa City, IA, USA; ^4^Department of Neuroscience, Carver College of Medicine, University of IowaIowa City, IA, USA; ^5^Department of Electrical Engineering, University of IowaIowa City, IA, USA

**Keywords:** brain MRI, label atlas, open-source, multi-modal, ITK, Huntingtons Disease, multi-atlas

## Abstract

The creation of high-quality medical imaging reference atlas datasets with consistent dense anatomical region labels is a challenging task. Reference atlases have many uses in medical image applications and are essential components of atlas-based segmentation tools commonly used for producing personalized anatomical measurements for individual subjects. The process of manual identification of anatomical regions by experts is regarded as a so-called gold standard; however, it is usually impractical because of the labor-intensive costs. Further, as the number of regions of interest increases, these manually created atlases often contain many small inconsistently labeled or disconnected regions that need to be identified and corrected. This project proposes an efficient process to drastically reduce the time necessary for manual revision in order to improve atlas label quality. We introduce the LabelAtlasEditor tool, a SimpleITK-based open-source label atlas correction tool distributed within the image visualization software 3D Slicer. LabelAtlasEditor incorporates several 3D Slicer widgets into one consistent interface and provides label-specific correction tools, allowing for rapid identification, navigation, and modification of the small, disconnected erroneous labels within an atlas. The technical details for the implementation and performance of LabelAtlasEditor are demonstrated using an application of improving a set of 20 Huntingtons Disease-specific multi-modal brain atlases. Additionally, we present the advantages and limitations of automatic atlas correction. After the correction of atlas inconsistencies and small, disconnected regions, the number of unidentified voxels for each dataset was reduced on average by 68.48%.

## Introduction

The study of human brain anatomy is important in clinical studies of normal brains as well as in studies on neurodegenerative disorders such as Huntingtons Disease (HD), Alzheimer's disease, and Parkinson's disease. A precise assessment of the volumetric characteristics of brain structures may provide a non-invasive means to monitor the treatment effects of clinical intervention. During the last decade, many studies have collected series of imaging data to better understand the brain. These studies of structural brain magnetic resonance imaging (MRI) have provided important understanding of healthy development (Sullivan et al., 2011; Treit et al., 2013; Herting et al., 2014), normal aging (Tang et al., 2001; Resnick et al., 2003; Scahill et al., 2003; Mungas et al., 2005; Risacher et al., 2010), and disease progression (Ahdidan et al., 2011; Tabrizi et al., 2012; Takahashi et al., 2012; Weiner et al., 2012; Li et al., 2015).

Atlas-based segmentation is a commonly used approach (Cabezas et al., 2011) that identifies regions of interests (ROI) by propagating atlas labeling to a target image. More recently, multi-atlas labeling approaches, instead of single-atlas labeling, have gained popularity for their superiority in segmentation quality (Kim et al., 2015). Naturally, the performance of this atlas-based segmentation largely depends on how well the atlas structures are defined and the similarity between the atlas and the research population.

Currently, there are limited atlas labels available in the field (Tzourio-Mazoyer et al., 2002; Mori et al., 2005). While manual identification of brain structures from MRI is considered a gold standard, the creation of a population or research-specific atlas is limited by its labor-intensive and time-consuming nature. Furthermore, if one chooses to use multi-atlas labeling approaches, which show increasing evidence of improved segmentation quality (Kim et al., 2015), manual approaches for label atlas creation often become impractical for ongoing research. The choice of atlases for brain research is therefore often limited to what is already available, even though there is a danger of bias when using unrepresentative atlases for the study of interest.

There has been a concern about the accuracy and consistency of manual traces for brain MRI structures in the neuroimaging community. Recently, using a collection of open-source tools called Open Atlas (Lorensen, 2015), we visually investigated and quantitatively confirmed that manually identified brain structures can be inaccurate because of the large numbers of small, disconnected regions (islands) that are biologically invalid. These inaccuracies of manually identified structures are mainly a result of the current limitations of the tracing environment, wherein experts segment convoluted three-dimensional structures within two-dimensional planes. These label atlas errors may have been underestimated previously since they are difficult to recognize in a two-dimensional display.

In this paper, we propose a prototype application for creating and/or improving a set of MRI label atlases by incorporating prior information as well as spatial heuristics with highly automated procedures. The primary goal of this project is to provide an efficient procedure that reduces the editing time to a few hours while providing valid segmentation results. To provide an efficient label-editing graphical user interface (GUI) in one easily accessible window, we utilized 3D Slicer, a free open-source tool for image visualization and computing (Fedorov et al., 2012). We further incorporated several useful editing tools provided in 3D Slicer, such as Editor (Pieper et al., 2014) and Markups (Aucoin, 2014), in addition to our newly developed procedures. This proposed application, LabelAtlasEditor, is freely distributed with 3D Slicer, and we expect the proposed approach to require less time for manual intervention in creating and/or improving MRI label atlases.

We demonstrate our label atlas correction approach by using a dataset of 20 subjects spanning a range of HD progression and evaluate the performance of our atlas correction tools. These subjects were selected from the PREDICT-HD dataset that contains pre-symptomatic gene-positive subjects collected during a 10-year time period (Paulsen et al., 2006). In this article, we will walk through the steps used for identifying the regions of interest with a semi-automated tool, detail the method used for automatically removing small disconnected regions of voxels, and demonstrating a technique for cleaning the regions of interest with prior probability maps.

## Materials and methods

In this section, the dataset used in this study is described. It is then followed by an illustration of the MRI pre-processing that we applied to our atlases. Finally, we detail a set of developed applications for label atlas generation and the proposed procedure for developing a reliable set of label atlases by using a series of automatic tools including those that we developed.

### Dataset

The HD label atlas candidates were carefully selected from the PREDICT-HD database. The 20 MRI subjects selected consisted of 10 males and 10 females between the ages of 28.4 and 68.1 years. These subjects span the HD disease index of control, low, medium, and high CAG (cytosine-adenine-guanine) repeat lengths. Scans were collected from three types of scanners: GE, Siemens, and Philips. All datasets are multi-modal with most containing multiple T1-weighted (T1-w) and T2-weighted (T2-w) images from repeated data sessions. Demographic variables (gender, age, and CAG repeat length) are reported in Table 1 and the imaging acquisition parameters for the scans are reported in Table 2. The T1-w, T2-w, initial label atlas, and corrected label atlas images for the 20 datasets are available through MIDAS (BRAINSTools, 2016).

**Table 1:** **HD Atlas set demographics**.

**Manufacturer**	**Gender**	**CAP**	**Age**	**Summary**	
GE	F	Med	28.4		
GE	F	Med	46.5	Manufacturer	
GE	F	High	56.4	GE	7
GE	F	High	58.0	Philips	6
GE	M	Low	31.3	Siemens	7
GE	M	Cont	36.0	Total	20
GE	M	High	40.5		
Philips	F	Med	36.0		
Philips	F	High	44.7	Gender	
Philips	F	Cont	47.3	Male	10
Philips	F	High	59.2	Female	10
Philips	M	Cont	43.6	Total	20
Philips	M	High	46.8		
Siemens	F	Low	36.4		
Siemens	F	Med	39.9	CAP	
Siemens	M	High	41.6	Control	4
Siemens	M	Med	41.9	Low	2
Siemens	M	High	51.6	Med	5
Siemens	M	High	55.6	High	9
Siemens	M	Cont	68.1		

**Table 2:** **HD Atlas MRI Imaging parameter summary (the magnetic field strength for all scans is 3T)**.

**Mod**	**Scanner**	**Scanner Type**	**TR (ms)**	**TE (ms)**	**TI (ms)**	**Thickness (mm)**	**Acq. Matrix**
T1	GE	Signa HDxt	6.524–7.816	2.796–3.004	450	1.0	[256, 256]
	Philips	Intera, Achieva	7.313–7.7	3.271–3.501		0.63, 1.1	[256, 256] [220, 218]
	Siemens	TrioTim, Allegra	2300	2.67–2.98	900	0.7, 0.75, 1.1	[256, 256] [320, 320]
T2	GE	Signa HDxt	3000–15,000	39.6–100.128		1.1, 1.4, 1.8	[256, 256] [288, 288]
	Philips	Intera, Achieva	2500	181.29–185.97		1.1	[220, 218]
	Siemens	TrioTim, Allegra	4800	354–433		0.7, 1.4	[256, 248] [256, 250] [256, 254]

Mod, Modality; TR, Repetition Time; TE, Echo Time; TI, Inversion Time; Acq. Matrix, Acquisition Matrix.

### Preprocessing

All repeated scans in one MRI session were processed together, i.e., repeated T1-w and T2-w MRI using the BRAINSTools suite (Pierson et al., 2011; BRAINSia, 2015). Experts rated the quality of each MRI to determine its suitability for further processing and then ordered the scans from high to low quality. MRI scans were then preprocessed using tools from the BRAINSTools suite. The preprocessing of MRIs consists of an AC-PC spatial alignment (Lu, 2010; Ghayoor et al., 2013), co-registration between T1-w and T2-w images, and multimodal bias-field correction (Kim and Johnson, 2013).

To create an initial set of label atlases for these 20 HD atlas candidates, we first automatically segmented label atlases by using the ANTs joint fusion algorithm (Wang et al., 2012; Wang and Yushkevich, 2013). The joint fusion algorithm was applied on our HD MRI atlas candidates by using 20 T1-w MRI with whole brain label atlases that were already available from Neuromorphometrics Inc. Note that we further incorporated white matter sub-parcellation by using FreeSurfer automatic segmentation so that our output incorporates both white and gray matter sub-parcellation. The outcomes of ANTs joint fusion with our white matter segmented Neuromorphometrics data were then ready for our label atlas correction procedure.

### Labelatlaseditor

We developed a user interface, within the infrastructure of the imaging software 3D Slicer, to visualize and efficiently edit label atlases. We used the open-source image processing toolkit SimpleITK (Lowekamp et al., 2013) for processing the label atlases. In this section, we describe the custom user interfaces, widgets, that we developed for correcting these brain structures: (1) The *Label Merge* widget allows a user to utilize a mask or a posterior probability map while merging the voxels of different labels in order to ensure that a voxel meets a user-defined minimum probability for a specific type, e.g., white matter or cerebrospinal fluid. (2) The *Label Suggestion* widget provides a list of candidate labels for a questionable group of voxels based on the neighborhood information attained from intensity images. (3) The *Automatic Dust Cleanup* widget automatically merges large amounts of small, disconnected regions to the most similar bordering label via the process employed in the Label Suggestion widget.

For convenience, we included the 3D Slicer widgets Editor and Markups within this all-in-one module to expedite the manual cleaning process when required. Assuming that one label represents a single biological structure or densely packed structures, small isolated regions should be examined for validity. The open-source software OpenAtlas can be used for providing an excellent three-dimensional (3D) visualization of the disconnected ROIs. This tool identifies errors that are difficult to visually recognize in two-dimensional slices, by placing a fiducial point on each disconnected region. Within the Markups widget, the user selects a target region from the fiducial points to view and easily modify by using Editor or one of the custom widgets that we developed.

#### Label merge widget

**Figure 1:**
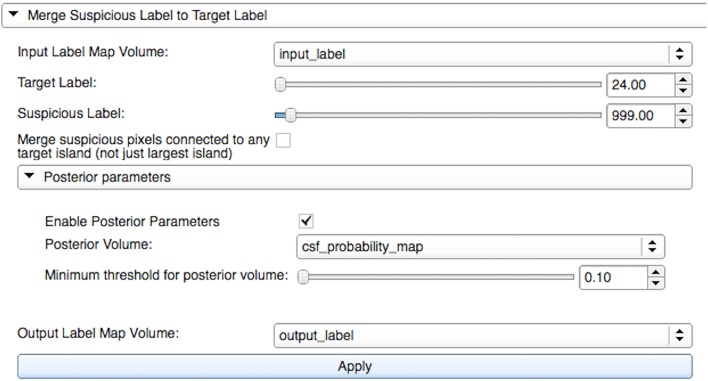
**Merge widget graphical user interface within LabelAtlasEditor**. The input parameters include: (1) *Input Label Map Volume* specifying the label image, (2) *Target Label* specifying the identification number of the target label to which new voxels are reassigned, (3) *Suspicious Label* specifying the identification number of the source label from which voxels are reassigned, (4) a check box to either reassign the source voxels contained within the largest island of connected voxels or within all islands of connected voxels, (5) *Posterior Volume* providing optional apriority information via a mask or a probability label map image for a specific type, (6) *Minimum Threshold* used for restricting the merging of voxels that do not meet this value in the *Posterior Volume* image, and (7) *Output Label Map Volume* specifying the output label image. In this figure, “input_label” voxels from the source label “999” (unsegmented) are merged with the target label “24” (cerebrospinal fluid-CSF) if they are within the largest connected island and have a corresponding value in “csf_probility_map” that is larger than the user-defined minimum value of “0.10.” The resulting label map is saved to “output_label.”

The Label Merge widget is useful for reassigning incorrectly defined islands surrounding well-defined structures. It autocorrects voxels from a source label to a target label at locations where the two are connected. Initially, a combined mask of the source and the target labels is created. Then, islands of connected voxels are identified. As shown in Figure 1, the user can choose to reassign the source voxels contained within the largest island of connected voxels or within all islands of connected voxels. Optional apriority information via a mask or a probability label map image for a specific type, e.g., white matter or cerebrospinal fluid, can be used for restricting the merging of voxels that do not meet the user-defined minimum value.

#### Label suggestion widget

**Figure 2:**
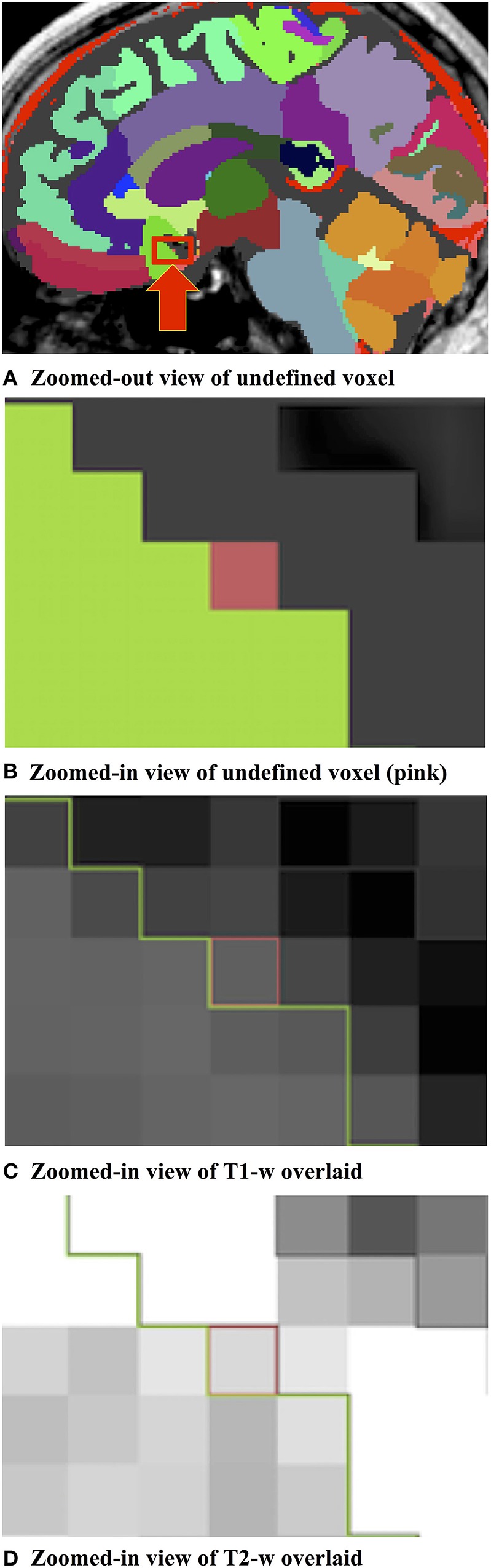
**(A)** Label image containing an ambiguous, undefined pixel within the red box indicated by a red arrow. **(B)** Zoomed-in example of an undefined pixel (shown in pink) at a border between two regions of interest illustrated by the “subcallosal” (green) and “CSF” (dark gray) labels. **(C)** Zoomed-in illustration of the T1-w pixel intensity values of the undefined pixel and the bordering labels. **(D)** Zoomed-in illustration of the T2-w pixel intensity values of the undefined pixel and the bordering labels.

**Figure 3:**
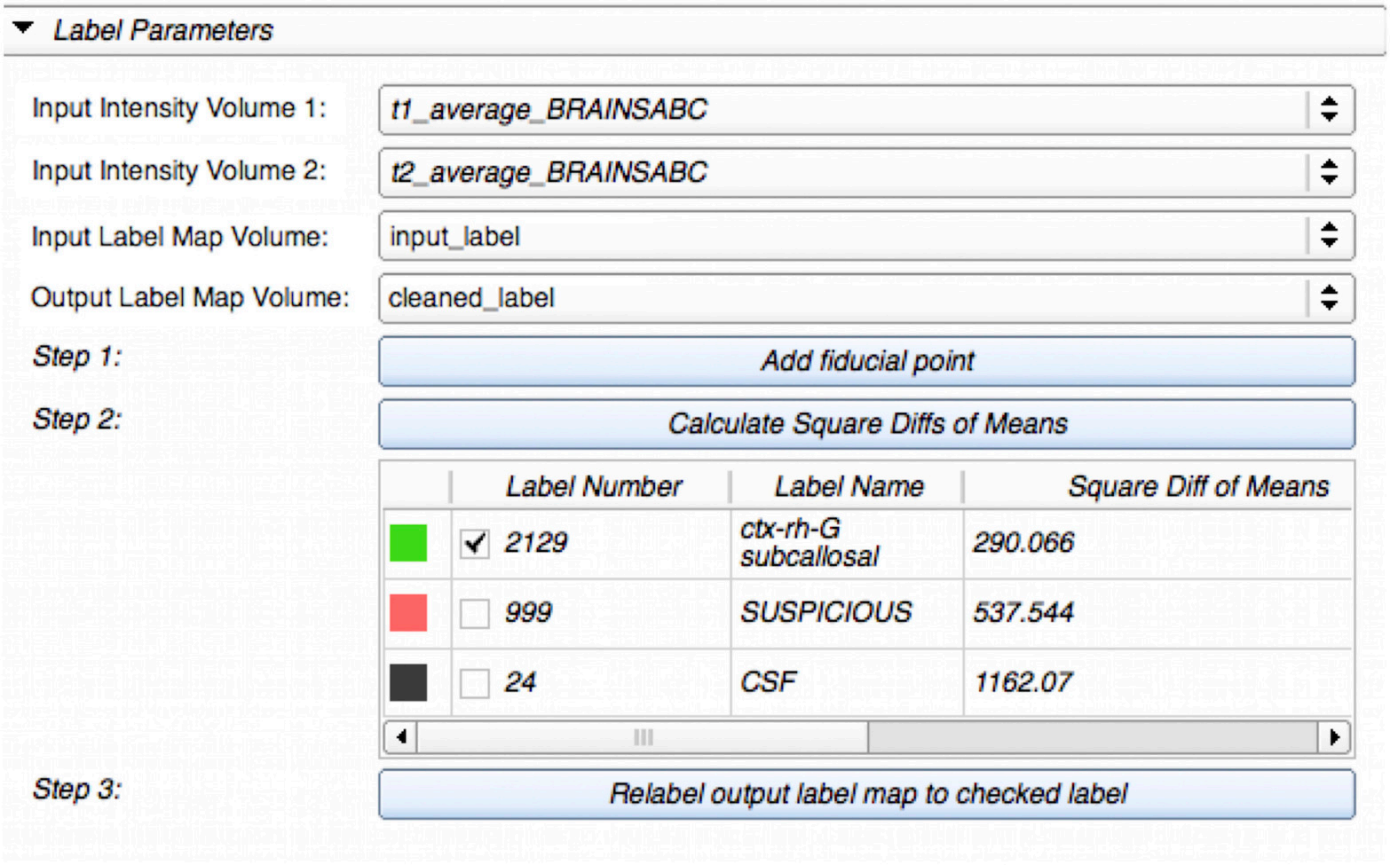
**Label Suggest widget's graphical user interface within LabelAtlasEditor**. The input parameters include: (1) *Input Intensity Volume 1* specifying the T1-w image, (2) *Input Intensity Volume 2* specifying the T2-w image, (3) *Input Label Map Volume*, and (4) *Output Label Map Volume*. This figure displays an example use-case in which a fiducial point was placed at the suspicious voxel (pink) displayed in Figure 2. The label “2129-subcallosal” (green) has been selected as the most similar adjacent region for the ambiguous region. The voxels within the ambiguous region are reassigned to the selected label in the *Output Label Map* via the *Relabel* button.

We developed a custom filter that suggests candidate labels for a questionable region. It is often difficult, even to experts, to visually assign a label for an ambiguous voxel, as illustrated in Figure 2. Our Label Suggestion widget provides a quantitative measure for each neighboring label to remove ambiguity for small regions such as those identified by OpenAtlas. As shown in Figure 3, the label candidate list is ordered by a similarity criterion computed for each label on the target region's border. The similarity metric that we used in this study is a distance measurement of the mean intensity per region per modality given by:

$$\begin{array}{l} d=\sqrt{\underset{For\;all\;image\;i}{\sum }{\left(T_{i}-B_{i}\right)}^{2}} \end{array}$$

where *T*_*i*_ is the mean intensity value of the target region for image modality *I* and *B*_*i*_ is the mean intensity value of a border label region for image modality *i*. The border label with the smallest similarity metric has the closest average intensity to the target region.

#### Automatic dust cleanup widget

**Figure 4:**
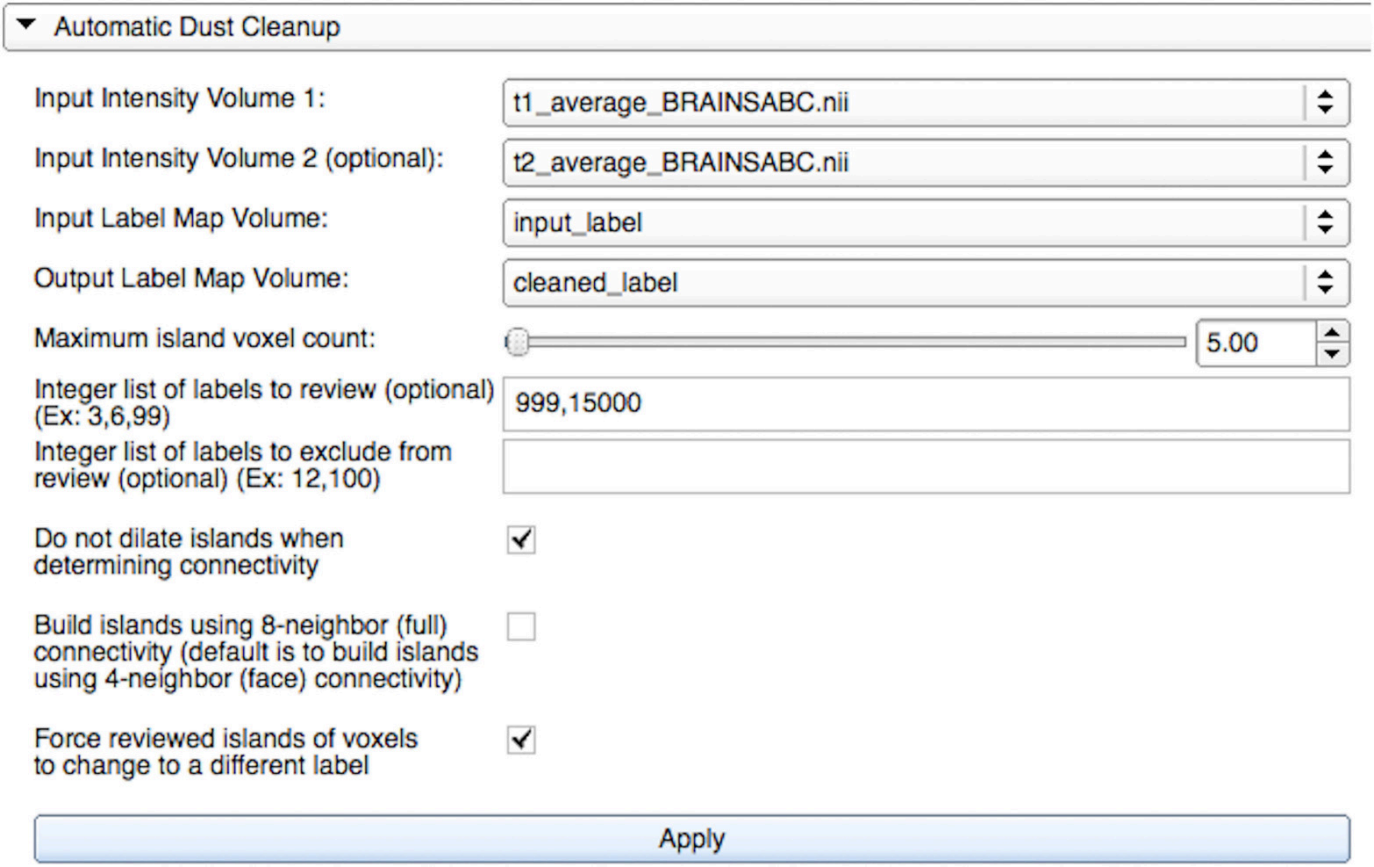
**Automatic Dust Cleanup widget's graphical user interface within LabelAtlasEditor**. The input parameters include: (1) *Input Intensity Volume 1* providing the T1-w image, (2) *Input Intensity Volume 2* providing the T2-w image, (3) *Input Label Map Volume*, (4) *Output Label Map Volume*, (5) the *Maximum Island Voxel Count* specifying the largest island size to correct, (6) *List of Labels to Review* identifying labels to correct, (7) *List of Labels to Exclude* identifying labels to exclude from correction, (8) a check box specifying either to dilate or not dilate islands when determining connectivity, (9) a check box specifying to build islands with full or face connectivity, and (10) a check box specifying to force an island label to change even if it is most similar to its own label. In this example, islands consisting of “5” or fewer voxels in the labels “999” and “15,000” will be reassigned to the most similar adjacent label. Islands will be built using face connectivity and islands in densely packed groups will be reassigned given that the mask is not dilated.

To further expedite the correction process and reduce manual interaction, we developed an automated process that reassigns small, disconnected islands of voxels, *dust*, by using the underlying process described in Section Label Suggestion widget. As illustrated in Figure 4, the user may define a list of labels to include or exclude from the correction. The algorithm will review all labels if either list is omitted. It proceeds through the label list by correcting the label image one label at a time. Within each label *l*, we increase the island size *s* from 1 to *S*, the user-defined maximum island voxel count. All islands of size *s* are named sequentially from *s*_1_ to *s*_*N*_ and are reviewed one by one.

It is possible for a label to consist of several densely packed islands of voxels; therefore, the proposed algorithm accounts for the proximity of a questionable island to the other bodies within that label. The goal is to correct islands in each label that are spatially isolated and therefore, more likely to be incorrectly identified. To identify isolated islands, we dilated the binary mask of label *l* by using the kernel radius calculated from the current island size, given that it is larger than one voxel. Dilation was not performed on islands containing only one voxel in order to remove all isolated single voxels. The kernel radius, *r*, was set to the radius of a sphere given volume *s*, the current island voxel count. That is,

$$r=\left\lceil \sqrt[{3}]{\frac{s}{\frac{4}{3}*\;}}\right\rceil .$$

Islands are built from the resulting binary mask for label *l* on the basis of the user-specified option for either four-neighbor (face) connectivity or eight-neighbor (face + edge + vertex full) connectivity. If the island size *s* is greater than one, the resulting image identifying islands was then masked by the original non-dilated mask for label *l* in order to create a label map based on the dilated label mask connections. Each island of size *s* is then merged with the neighboring label containing the most similar average pixel intensity values from the provided intensity images, as described in Section Label Suggestion widget. The user can specify to force the island label to change even if it is most similar to its own label. This cleaning process can also be performed on the command line with the same input parameters.

### Proposed procedure using 3D slicer with our label correction widgets

This section demonstrates the application of LabelAtlasEditor and describes the process used to clean atlases in the PREDICT-HD dataset. The tools utilized in this study are available in 3D Slicer Extensions Manager and the source code manager Github (Forbes, 2016).

#### Stage 1: addition of new regions using editor

We identified and added new regions of interest to label maps by using the semi-automated Editor tool within 3D Slicer. The new regions of interest include the optic chiasm, Dura, and pineal gland. We then identified and corrected obvious issues such as islands incorrectly located outside of the brain region.

#### Stage 2: automatic cleanup of unidentified dust using the automatic dust cleanup widget

One of the goals of the atlas cleanup process is to reduce the number of unidentified voxels classified as the “suspicious” label. To assign appropriate labels to the dust in this label, we used the Automatic Dust Cleanup widget to change the islands of six voxels or less to the most similar bordering label. We noted that islands greater than seven voxels often needed to be split into more than one label and were therefore excluded from this process. In order to break the unidentified voxels into smaller groups, we used the four-neighbor, instead of the eight-neighbor, connectivity option when building islands. To remove all unidentified dust particles, we selected the option to force label reassignment. This was performed without dilation in order to maximize the number of islands cleaned. Since ideally we would like to remove all islands of suspicious pixels, we did not dilate the mask. This allowed the widget to change small islands spatially close to other unidentified islands.

#### Stage 3: semi-automated cleanup with the merge widget and editor tools

In this stage, we used the Editor tools to reassign larger unidentified islands to the correct label. Further, some label atlases contained excess venous blood beyond the brain region. To correct this, we utilized the Merge widget with a venous blood mask based on intensity ranges from both the T1-w and the T2-w images. With this process, we efficiently reassigned these voxels to the background label.

#### Stage 4: reassign isolated dust particles for most labels using the automatic dust cleanup widget

We used the Automatic Dust Cleanup widget to change the islands of five voxels or less to the most similar bordering label. In this stage, our goal was to automatically reassign the isolated dust particles observed in many brain structures. Labels with viable isolated islands, such as the cerebrospinal fluid (CSF), were excluded from this stage. We selected the eight-neighbor connectivity option when building the islands and the option to force label reassignment. The process was performed with dilation to limit automatic label reassignment of viable island clusters.

## Results

**Figure 5:**
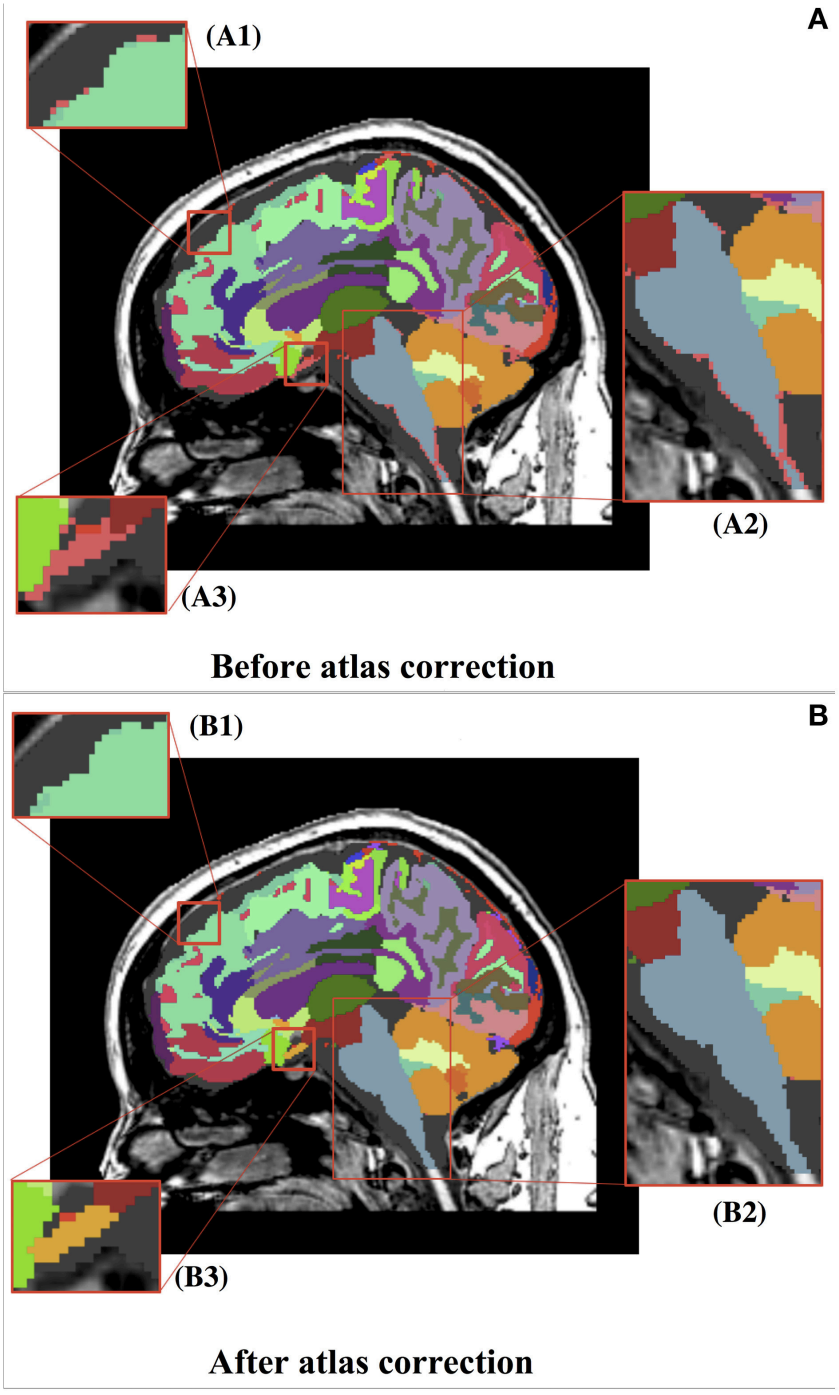
**Before (A) and after (B) representation of the whole brain atlas and zoomed-in views of atlas corrections**. **(A)** Thousands of undefined voxels occur at label borders (pink) because of partial volume effects. **(B)** The automatic dust removal process assigned previously undefined voxels and isolated islands. **(B1)** Displays the reassignment of unsegmented voxels (pink) from **(A1)** to the superior frontal label (teal). **(B2)** Displays the reassignment of unsegmented voxels (pink) from **(A2)** to the brainstem label (blue). **(B3)** Displays the reassignment of unsegmented voxels (pink) from **(A3)** to a new, manually identified label for the optic chiasm (yellow). It can be seen that the overall segmentations were maintained.

**Figure 6:**
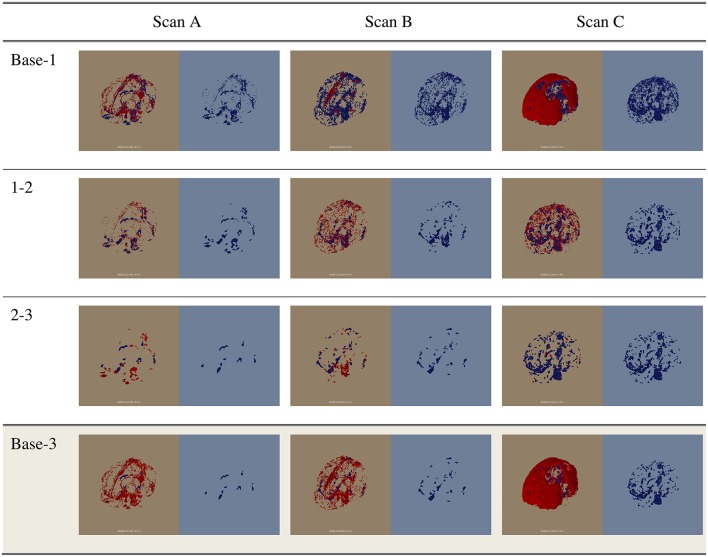
**The change in the unidentified voxels through the first three correction stages (Base-1, 1-2, and 2-3) and the change from the base to Stage 3 (Base-3)**. Scans A, B, and C are examples of datasets containing low, average, and high amounts of unidentified voxels. For each image, the red voxels in the left half of the image (brown) indicate the voxels removed in this stage. The voxels shown in blue in the right half of the image (blue) are the remaining unidentified voxels at each stage.

**Figure 7:**
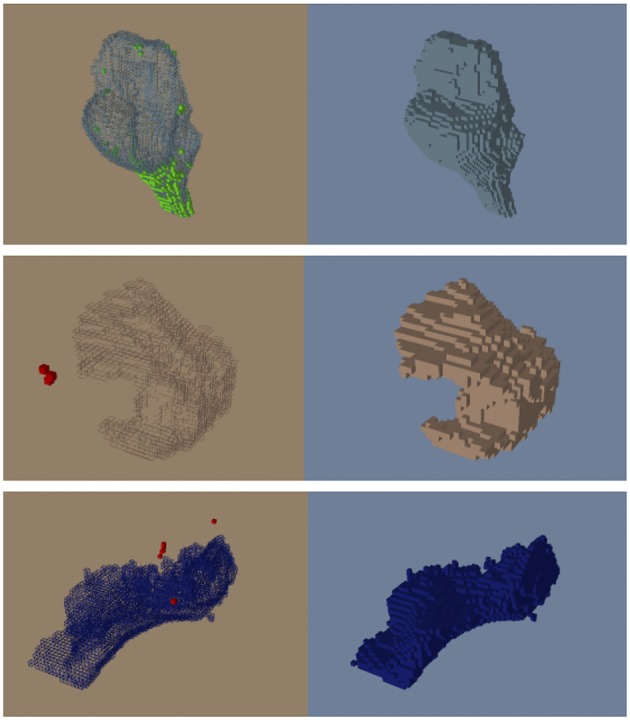
**Before (left) and after (right) the automatic dust cleaning process**. Voxels that are added (green) and removed (red) are color-coded using OpenAtlas. **(Top)** Example of voxels spatially adjacent to the *brainstem* (green) reassigned to the *brainstem* label. **(Middle)** Example of isolated voxels (red) removed from part of the *inferior frontal gyrus*. **(Bottom)** Example of isolated voxels (red) removed from the *lingual* label.

The proposed atlas generation approach successfully identified and reassigned large numbers of undefined and isolated islands to a proper structure based on available prior information and morphometric operations. The dust removal process successfully decreased the plethora of small, isolated islands often scattered throughout the image. As illustrated in Figure 5, the overall brain structure definitions were maintained. Figure 6 shows the progress of our approach for undefined regions in three example datasets. Figure 7 demonstrates the results of the automatic assignment of small, disconnected islands of voxels from most labels in the atlas.

**Figure 8:**
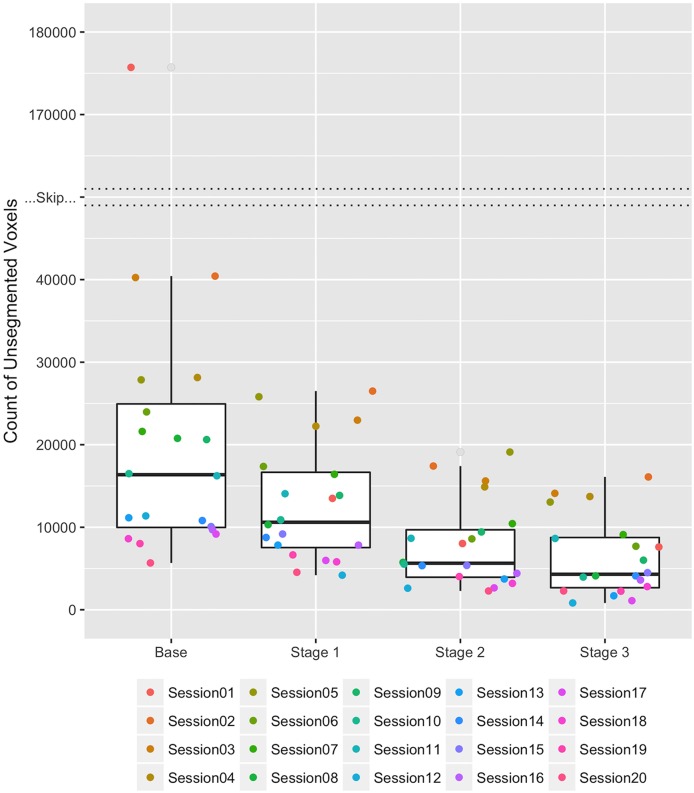
**Boxplots of the count of undefined voxels remaining during each stage of correction for the 20 HD datasets**. Each of the 20 datasets is represented by a consistently colored dot in each stage of correction. The “Skip” represents a break in the y-axis between 40,000 and 170,000 unsegmented voxels necessary due to an extreme outlier in the Base stage. The count of undefined voxels decreased over the stages Base, 1, 2, and 3 as follows: (Maximum) 175,715, 26,495, 19,111, and 16,095; (Third Quartile) 24,942, 16,652, 9683, and 8759; (Median) 16,364, 10,608, 5639, and 4305; (First Quartile) 9980, 7538, 3952, and 2676; and (Minimum) 5674, 4188, 2282, and 828.

The average number of undefined voxels was reduced at each stage of the correction procedure as seen in Figure 8. The median counts of undefined voxels in the stages Base, 1, 2, and 3 were 16,364, 10,608, 5639, and 4305, respectively. There is one large outlier in the Base stage with 175,715 undefined voxels. This number was reduced to 13,498 after Stage 1, near the median count of 10,608 for label atlases in Stage 1. The majority of voxels for this outlier were merged with the background label because they were outside of the brain region. The average percent reduction in undefined voxels for each dataset was 68.48% with a standard deviation of 14%. The maximum percent reduction in undefined voxels was 95.67% (this reduction occurred for the outlier) and the minimum percent reduction was 46.76%.

The semi-automatic reassignment of unidentified voxels to new labels and the correction of atlas issues in Stage 1 took between 30 min and 1.5 h depending on the quality of the initial atlas segmentations. On a quad-core Intel Xeon machine, the automatic cleaning process for the undefined voxels in Stage 2 took an average of 20.2 min to complete with a standard deviation of 7.7 min. Then, the semi-automatic reassignment of larger unidentified islands to the correct label and further correction of atlas inconsistencies took between 30 min and 1.5 h depending on the quality of the atlas segmentation. Finally, the automatic cleaning process for most labels in Stage 4 took an average of 12.2 min to complete with a standard deviation of 1.3 min. The total time for the cleaning process (including all stages) ranged from 1.5 to 4 h per dataset. Considering there are thousands of islands in a dataset and manual reassignment of each island takes approximately 45 s, the automatic cleaning process substantially improved editing time. The manual and semi-automatic revision stages are optional, but recommended if the datasets will be used as template atlases. No effort was made to optimize computational time for the Python implementation of the automatic cleaning tool.

## Discussion

This work described the creation of a multi-atlas dataset for the entire brain region that spans a range of HD progression and MRI scanners. These atlases are expected to improve the future atlas-based MRI analysis on HD. Our tool was utilized to decrease the editing time for the 20 considered atlases by providing automatic and semi-automatic reassignment of islands. After the correction of atlas inconsistencies and small, disconnected regions, the number of unidentified voxels for each dataset was reduced on average by 68.48%. Additionally, new regions of interest were efficiently identified and included in the atlases.

The Automatic Dust Clean Up widget was designed for use in correcting small, disconnected islands and may not be accurate when applied to larger islands. As the islands increase in size, the probability that the voxels belong to more than one label increases and it is more appropriate to examine the islands voxel by voxel.

As this is a limitation, future work may include developing a method to reassign voxels in larger islands individually starting at the perimeter and working in to the center. Additionally, although the computational time for the Python implementation of the automatic cleaning tool is not a limitation of this study, future work may include optimizing the implementation of this tool.

The LabelAtlasEditor offers an efficient, user-friendly method for editing label segmentations. The relative ease of atlas manipulation simplifies the correction of atlases and the tool is flexible enough to be used for anatomical regions other than the brain. LabelAtlasEditor has broad potential applications and is available for free download in the Extension Manager and for use as part of 3D Slicer.

## Author contributions

JF, RK, HJ, and JP all contributed to the paper.

## Funding

This study was funded by multiple grants: Huntington's Disease Society of America (Human Biology Project Fellowship), BRAINS (R01 NS050568), Validation of Structural/Functional MRI Localization (R01 EB000975), 3D Shape Analysis for Computational Anatomy (R01 EB008171), Neurobiological Predictors of HD (R01 NS040068), Cognitive and Functional Brain Changes in Preclinical HD (R01 NS054893), Algorithms for Functional and Anatomical Brain Analysis (P41 RR015241), Enterprise Storage in a Collaborative Neuroimaging Environment (S10 RR023392), Core 2b HD (U54 EB005149), and NIPYPE (R03 EB008673).

### Conflict of interest statement

The authors declare that the research was conducted in the absence of any commercial or financial relationships that could be construed as a potential conflict of interest. The reviewer AL and handling Editor declared their shared affiliation, and the handling Editor states that the process nevertheless met the standards of a fair and objective review.
